# Fabrication and Evaluation of Isomalt-Based Microfibers as Drug Carrier Systems

**DOI:** 10.3390/pharmaceutics17081063

**Published:** 2025-08-15

**Authors:** Andrea Kovács, Bálint Attila Kecskés, Gábor Filipszki, Dóra Farkas, Bence Tóth, István Antal, Nikolett Kállai-Szabó

**Affiliations:** 1 Department of Pharmaceutics, Semmelweis University, Hőgyes E. Str. 7–9, 1092 Budapest, Hungarykecskes.balint@stud.semmelweis.hu (B.A.K.); filipszki.gabor@stud.semmelweis.hu (G.F.); toth.bence@semmelweis.hu (B.T.); 2 Center for Pharmacology and Drug Research & Development, Semmelweis University, 1085 Budapest, Hungary

**Keywords:** isomalt, melt spinning, microfiber, drug delivery system, drug carrier, cotton candy method, floss technology, FlashDose^®^ technology, orally disintegrating tablets (ODTs)

## Abstract

**Background/Objectives**: The melt-spinning process has seen limited application in the pharmaceutical industry. However, nano- and microfibrous structures show significant potential for novel drug delivery systems, due to their high specific surface area. To facilitate broader adoption in pharmaceutical technology, critical parameters influencing fiber quality and yield must be investigated. In this study, we aimed to develop an isomalt-based microfibrous carrier system for active pharmaceutical ingredients. **Methods**: The effects of different isomalt compositions—specifically, varying ratios of GPS (6-*O*-α-d-glucopyranosyl-d-sorbitol) and GPM (1-*O*-α-d-glucopyranosyl-d-mannitol)—as well as key process parameters, were systematically investigated to optimize fiber formation. The prepared fibers underwent different treatments. Morphological changes were monitored with a microscope, and microstructural changes were studied using a differential scanning calorimeter and X-ray diffractometer. The macroscopic behavior of the fibers was evaluated by image analysis under monitored conditions. **Results:** Statistical analysis was used to determine the optimal setting to produce isomalt-based fibers. We found that storage over ethanol vapor has a positive effect on the stability of the fibers. We successfully prepared ibuprofen sodium-containing fibers that remained stable after alcohol treatment and enabled drug release within 15 s. **Conclusions:** It was found that the applied GPS:GPM isomalt ratio significantly influenced fiber formation and that storage over ethanol positively influenced the processability and stability of the fibrous structure. An isomalt-based microfibrous system with advantageous physicochemical and structural properties was successfully developed as a potential drug carrier. The system is also resistant to the destructive effects of ambient humidity, enabling preparation of suitable dosage forms.

## 1. Introduction

Isomalt has been extensively utilized for decades, particularly in the formulation of sugar-free or reduced-calorie confectionery items such as toffees, hard candies, chocolates, chewing gums, and baked goods [1,2,3,4]. Beyond its role in food applications, its relevance in the pharmaceutical field has grown steadily, owing to its versatility across various dosage forms and its suitability for sugar-free formulations. Consequently, isomalt is recognized as an official excipient in several pharmacopeias [5,6].

The production of this excipient consists of two main steps. Firstly, an enzymatic transglucosidation process converts the non-reducing sucrose molecule into the reducing molecule α-d-glucopyranosyl-1,6-d-fructose, generically called isomaltulose. The 1-6 bond formed between the glucose and fructose molecule is much more stable than the 1-2 bond in the sucrose molecule, making isomaltulose more resistant to acids and microbial agents. The next step is catalytic hydrogenation (with Raney nickel), which results in the formation of the stereoisomers 6-*O*-α-d-glucopyranosyl-d-sorbitol (1,6-GPS) and 1-*O*-α-d-glucopyranosyl-d-mannitol (1,1-GPM) in almost equal proportions. GPM crystallizes into two molecules with water, while GPS crystallizes in anhydrate form [7,8]. The ratio of GPS to GPM can be varied by a special crystallization process. As a result, the GPS-rich end product has better water solubility than the GPS-poor end product [5].

From a pharmaceutical perspective, isomalt exhibits several advantageous properties compared to sucrose. In addition to being non-cariogenic (tooth-friendly) and suitable for diabetic patients, it exhibits lower hygroscopicity [9,10]. It also has half the sweetening power of sugar and has no unpleasant taste or aftertaste [8].

Isomalt is a pharmaceutical excipient suitable for use in various dosage forms, commercially available in different particle sizes and with varying GPS and GPM ratios. Langer produced solid dispersions containing various active ingredients and used four types of sugar alcohol as excipients in the study, with results showing that isomalt was the most suitable carrier [11]. Isomalt has been shown to enhance the solubility of certain active pharmaceutical ingredients (APIs) [12]. As an inert pellet core, it serves as a seed for multiparticulate systems [13,14,15,16]. Isomalt can be used for the production of oromucosal films due to its sweet taste [17,18]. It is suitable for the development of 3D-printed printlets [19,20]. Tuderman showed that isomalt is suitable as a protein-stabilizing excipient in lyophilization [21]. Isomalt is also suitable as an excipient for granule formation. In a 2011 publication, Sáska et al. performed agglomeration using isomalt (galenIQ™ 801) and water without any additional binders [22]. Several research groups have successfully used isomalt to produce co-processed excipients by combining it with other materials [23,24]. Several studies have been published in which authors have investigated the compressibility of isomalt [25,26,27]. The commercially available aggregated isomalt is suitable for direct compression due to its good granule flowability and good compressibility. It can be used for the production of tablets, chewable tablets, and—when combined with suitable excipients—mini-tablets and orally dispersible tablets (ODTs) [28,29,30]. Unlike conventional direct compression methods used in ODT production, the patented FlashDose^®^ technology employs a preliminary melt-spinning process to create a fibrous matrix, which is then formed into rapidly disintegrating tablets. Developed by Fuisz Technologies, this approach uses sugars such as sucrose, dextrose, lactose, or fructose as the spinning material. To condition the amorphous fibrous structure and ensure its stability, ethanol is sprayed onto the cotton candy-like fibers. The resulting fibers exhibit good adhesion and improved flow properties, making the fibrous structure suitable for the production of orodispersible tablets [31,32,33,34].

In this manuscript, we have investigated a novel use of isomalt. The applicability of different commercially available isomalt compositions with varying GPS and GPM ratios for melt fiber formation was investigated, as well as the stability of the fibers. For optimal production, a two-variable three-stage factorial design was applied to investigate the effect on yield of two main independent variables used in pre-production. In order to improve the pharmaceutical processability and environmental stability of the fibers, ethanol vapor treatment was employed. Unlike the previously described patented method involving direct ethanol spraying, our approach introduces a novel strategy by storing the fibers in an ethanol-saturated vapor environment. By leveraging the high specific surface area of the fibers, this method allows for uniform ethanol exposure while avoiding local dissolution or deformation that can occur with liquid application. To our knowledge, the use of ethanol vapor for fiber treatment in this context has not been previously reported, making this a potentially advantageous and innovative alternative.

## 2. Materials and Methods

### 2.1. Materials

For the comparison, two types of isomalt were used as raw materials and as starters for fiber-forming and preparation of melted samples, specifically galenIQ^TM^ 720 and galenIQ^TM^ 721 (BENEO-Palatinit GmbH, Mannheim, Germany). Currently, only two types of isomalt with different GPS:GPM ratios are commercially available from the manufacturer for pharmaceutical use. The properties of the isomalt types used are described in Table 1, based on the manufacturer’s specifications [35]. As a reference, crystalline saccharose was chosen (Molar Chemicals Ltd., Halásztelek, Hungary), and for the alcoholic treatment of the prepared fibers, ethanol (Molar Chemicals Ltd., Halásztelek, Hungary) was used. The model drug was ibuprofen sodium (I1892-500; Sigma-Aldrich; Supelco, Bangalore, India).

### 2.2. Methods

#### 2.2.1. Fiber Formation from Isomalt or Sucrose

For fiber formation, a cotton candy machine (MikaMax^®^ CCM-500, Ningbo, China) was used, which is based on the melt-spinning process. The device was modified to control the applied temperature and the rotational speed. To eliminate the difficulties of fiber formation due to the difference in the particle size of the applied isomalts, melted amorphous samples (solid dispersions) were first prepared, which were further used in the fiber-forming process. The device was heated up to the desired temperature before the fiber-forming process (pre-warming step, for at least 10 min), after which the isomalt was fed into it and the desired rotational speed was set. In order to determine the optimal process parameters, the effect of the modified device settings on fiber formation was investigated. The rotational speed was measured with a non-contact tachometer (Voltcraft^®^ DT-30LK, Conrad Electronic International GmbH&Co.KG., Hirschau, Germany), and the temperature with an infrared thermometer (Voltcraft^®^ IR 650-16D, Conrad Electronic International GmbH&Co.KG., Hirschau, Germany). Thermal imaging was performed using a thermal camera (Voltcraft^®^ WBS-220, Conrad Electronic International GmbH&Co.KG., Hirschau, Germany). For the preparation of sucrose-based cotton candy in the traditional sense, the above cotton candy machine was used without a toroid.

#### 2.2.2. Optimizing the Process Parameters: Statistical Analysis

To study fiber formation and determine the optimal process parameters, the effects of rotational speed and applied temperature on fiber yield were investigated. For this purpose, a two-factorial, three-level experimental design was used. The yield of the produced fibers was calculated using Equation (1):

(1) $$Yield\%\left(\%;\frac{w}{w}\right)=\frac{w_{fibers}}{w_{input}\;}\times 100$$

where *w_fibers_* and *w_input_* represent the weight of prepared fibers and the amount of the isomalt that was placed into the heated rotating spinning head, respectively.

The two independent factors as well as their three levels are shown in Table 2.

The effects of the independent variables (*x*_1_ and *x*_2_) on response *y* were modeled by polynomial Equation (2):

(2) $$y=b_{0}+b_{1}x_{1}+b_{2}x_{2}+b_{11}x_{1}^{2}+b_{22}x_{2}^{2}+b_{12}x_{1}x_{2}$$

where *y* is the response; *b*_0_, *b*_1_, *b*_11_, *b*_2_, *b*_22_, and *b*_12_ are coefficients; and *x*_1_ and *x*_2_ are factors.

The independent variables *x* (*x*_1_: applied process temperature and *x_2_*: rotational speed of the heatable spinning head) and parameters *b* mark the coefficients characterizing the main (*b*_1_, *b*_2_), the quadratic (*b*_11_, *b*_22_), and the interaction effects (*b*_12_). The main effects represent the average outcome when varying one factor at a time. The interaction coefficient determines how the response (*y*) changes when the two independent factors are changed simultaneously. The quadratic polynomial terms are included to investigate nonlinearity. Statistical analysis was performed using TableCurve 3Dv4.0 (Systat Software Inc., London, UK). In each case, three parallel measurements were carried out at the same relative humidity (RH) and ambient temperature (27 °C, RH = 40%), detected by a datalogger (Sender Temperatur Luftfeuchtigkeit, TFA Dostmann, Ottersberg, Germany). From the weight (5.00 g) of the melted isomalt placed in the apparatus and the weight of the prepared fibers, the yield (%) was determined.

#### 2.2.3. Preparation of Ibuprofen Sodium-Loaded Isomalt-Based Fibers

**Figure 1:**
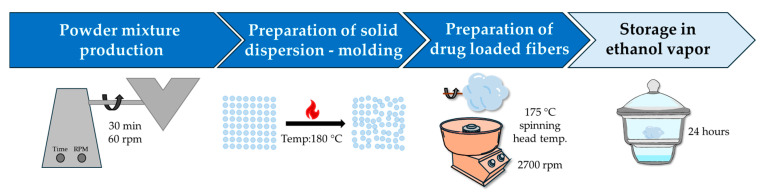
Schematic diagram of the production of ibuprofen sodium-containing isomalt-based fibers.

A homogeneous powder mixture was prepared for the production of fibers containing the active ingredient, consisting of 15% *w/w* ibuprofen sodium and isomalt (1:1; GPS:GPM). An amount of 150 g of the powder mixture was blended using a V-blender (CWV type; Xinxiang Chenwei Machinery Co., Ltd.; Xinxiang; China) for 30 min at 60 rpm. The fiber formation was then carried out as described in Section 2.2.1. Fibers containing the active ingredient were prepared under the optimal setup parameters determined from the statistical analysis. Figure 1 shows a schematic diagram of the process carried out on a laboratory scale. The ibuprofen sodium content in the isomalt-based fibers was determined by UV/VIS spectrophotometry. For this purpose, samples were taken from 10 different randomly chosen points of the fibrous mats and dissolved in phosphate buffer (pH = 6.8 ± 0.05) at room temperature. The drug concentration of the solutions was determined using an Agilent 8453 UV/VIS spectrophotometer (Agilent Technologies, Waldbronn, Germany) at 222 nm.

#### 2.2.4. Storage Conditions

The produced fibers were placed in two different exsiccators for 24 h. One exsiccator was saturated with air, and the other with 96% ethanol vapor, both at room temperature (25 °C ± 3 °C; Trotec BL30, Trotec GmbH & Co. KG, Heinsberg, Germany).

#### 2.2.5. Characterization Methods

##### Microscopic Analysis

To study the morphology, surface, and fragmentation after ethanol treatment, SEM images were recorded. The fibers (fresh, EtOH-treated) were fixed with conductive double-sided carbon adhesive tape and sputter-coated with gold for 60 s under argon atmosphere using a JEOL JFC-1200 Fine Coater (JEOL Ltd., Tokyo, Japan) to prevent electrostatic charging. Scanning electron microscopy (SEM) analysis was then performed using a JEOL JSM-6380LA microscope (JEOL Ltd., Japan) under high vacuum conditions, with a working distance of 10–15 mm and an accelerating voltage of 15 kV. To determine the fiber diameters, a Keyence VHX-970 digital microscope (Keyence, Japan) was used. For each sample investigated, the diameters of 100 fibers were measured. Image analysis was performed using ImageJ software (version 1.46; NIH, Bethesda, MD, USA), based on measurements of 10 fibers taken from 10 subsamples collected from random regions of the fibrous mats.

##### Macroscopical Morphology Monitoring

Changes in the macrostructure of the different fibrous samples (sucrose and isomalt fibers—with and without 24 h ethanol treatment) were monitored over time by taking photographs from top and side views with a camera (Olympus Stylus TG-4 digital camera, Olympus Corp., Tokyo, Japan). The fiber samples were subjected to simultaneous testing and evaluated following exposure to ambient conditions for 0, 30, 45, 60, 90, 120, 240, and 360 min, as well as after 24 h. For each type of sample, three parallel measurements were performed. During the testing period, the temperature and the relative humidity (average: 26.5 ± 1.8 °C, 44.5 ± 1.8% RH) were recorded with a climate data logger (Trotec BL30, Trotec GmbH & Co. KG, Heinsberg, Germany). The area was determined from top-view images, while the maximum height of the fibrous structure was assessed from side-view images using image analysis. Image processing and measurements were carried out using Fiji (ImageJ, open-source image analysis software). At each time point, three images were analyzed, and the results are reported as the mean ± standard deviation.

##### Differential Scanning Calorimetry (DSC) Measurements

To examine the phase transitions and thermoanalytical behavior of the solid samples (raw materials, freshly prepared fibrous samples, and ethanol-treated samples), differential scanning calorimetry (DSC) measurements were carried out using a Seiko Exstar 6000/6200 apparatus (Seiko Instruments Inc., Chiba, Japan). Quantities of 5–10 mg of solid samples were accurately weighted into aluminum pans. The thermal analysis was carried out from 6 °C to 210 °C with a heating rate of 10 °C/ min, under air atmosphere.

##### X-Ray Diffraction (XRD) Measurements

Diffraction patterns were recorded using a PANalytical X’Pert^3^ Powder diffractometer (Malvern Panalytical B.V., Almelo, The Netherlands) with Cu Kα radiation (λ = 1.5406 Å), operating at 45 kV and 40 mA. Measurements were performed in reflection mode over a 2θ range of 5°–40°, with a step size of 0.0084° and a counting time of 100 s per step. The sample holder was spun at 1 s^−1^ during data acquisition. Incident beam optics were as follows: programmable divergence slit with 15 mm constant irradiated length, and anti-scatter slit at fixed 2°. Diffracted beam optics consisted of an X’Celerator Scientific ultra-fast line detector with a 0.02 Soller slit and programmable anti-scatter slit with a 15 mm constant observed length. Samples were measured using “Sample Holder for Manually Prepared Powder Sample” (PW1811/16). The powder and fibrous samples were weighed into the sample holder and XRD measurements were performed. For the analysis of the melted samples (solid dispersions), the powder materials were measured directly into the sample holder, heated up to the melting temperature together with the sample holder, and subsequently cooled, which allowed the gap-free filling of the sample holder. Measurement data were collected by PANalytical Data Collector Application, version 5.5.0.505 (Malvern Panalytical B.V., The Netherlands).

##### In Vitro Drug Release Test

The dissolution test, performed according to Method 2.9.3. (Dissolution Test for Solid Dosage Forms) of the 11th Ph. Eur., was carried out using a rotating paddle apparatus (Hanson Vision^®^ Elite 8™, Hanson Research Corp., Chatsworth, CA, USA) with offline UV detection (Agilent 8453 UV/VIS spectrophotometer, Agilent Technologies) [36]. The parameters of the applied drug release method were as follows: 900 mL of pH 6.8 phosphate buffer as the dissolution medium, paddle rotation speed of 100 rpm, and temperature of 37.0 ± 0.5 °C. During the test, 5 mL samples were withdrawn at predetermined time points and filtered through a 10 µm filter (Hanson Research Corp.). The volume of the withdrawn samples was replaced with an equal volume of pre-heated dissolution medium. The active ingredient content was determined as described in Section 2.2.3. The dissolution test was performed in triplicate.

## 3. Results

### 3.1. Fiber Formation and Process Parameter Optimization

A commercially available cotton candy machine, modified for this purpose, was used to produce fibers from isomalt using the melt-spinning technique.

**Figure 2:**
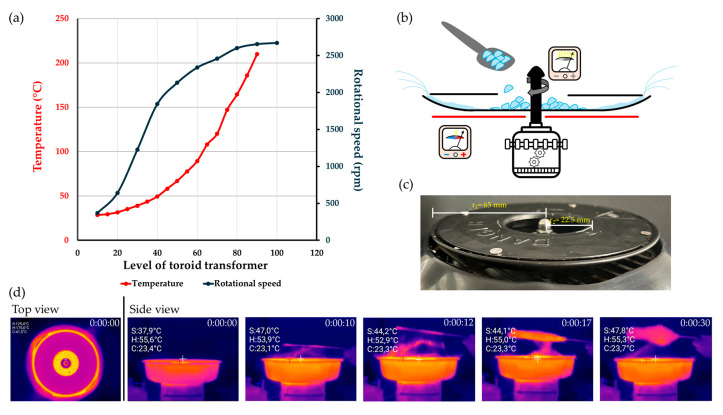
(**a**) Adjusting the setting parameters of the fiber-forming device; (**b**,**c**) schematic drawing and photo of the heated spinning head; (**d**) thermal camera images.

The second figure shows the temperature and RPM values for the different toroid levels. Figure 2b,c depicts heated spinning heads, while thermal camera images are also presented in the figure. The top view in Figure 2d shows the heated head and the fiber-forming process. Under conditions where fiber formation was successful, isomalt with a 1:1 GPS:GPM ratio, specifically galenIQ™ 720, was found to be the more suitable of the two types of isomalt for fiber production. The experimental design described in Section 2.2.2 was used to produce fibers from isomalt with different GPS:GPM ratios. According to the experimental plan, fiber samples were prepared using nine different setup parameters for each type of isomalt. The actual values of the parameters used during preparation, namely, temperature and speed, are listed in Table 3. To prevent large differences in the magnitudes of the two independent variables from distorting the coefficient estimates in the statistical analysis, we applied a commonly used coding method from the literature, also presented in Table 3 [37,38,39]. The yields obtained under the different setting parameter conditions (rpm, T of rotating head) are shown in Table 3 and serve as the basis for statistical analysis.

The effect of the two independent variables on the yields is shown by the response equations (Equations (3) and (4)) at a 95% significance level, alongside which the regression models for the different types of isomalt are also presented:

(3) $$1:1;\;GPS:GPM\;\;\;\;\;\;\;\;\;\;\;\;\;\;\;\;y\left(\%\right)=38.061-15.692x_{1}+22.708x_{2}\;\;\;\;\;\;\;\;\;\;\;\;R:0.971$$

(4) $$3:1;\;GPS:GPM\;\;\;\;\;\;\;\;\;\;\;\;\;\;\;\;y\left(\%\right)=22.858-10.245x_{1}+22.562x_{2}\;\;\;\;\;\;\;\;\;\;\;\;R:0.987$$

The results of the statistical analysis of the fitted models are presented in Table 4.

**Figure 3:**
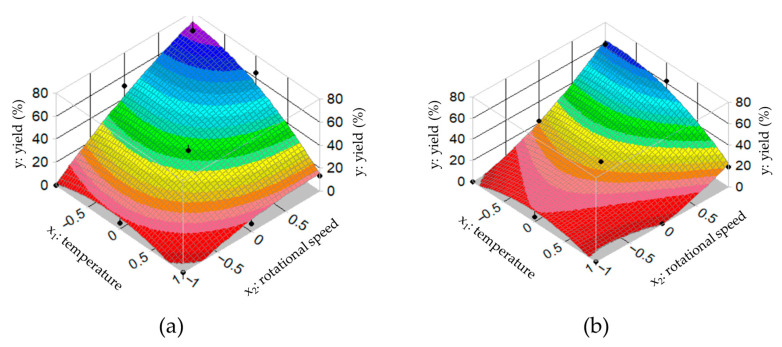
Surface plot of the effect of independent variables (T, rpm) on the production rate of (**a**) galenIQ^TM^ 720 (1:1; GPS:GPM) and (**b**) galenIQ^TM^ 721 (3:1; GPS:GPM)-based fiber formation.

Figure 3 illustrates the effects of the independent variables (temperature and speed) on the fiber yield.

For the production of fibers containing ibuprofen sodium, the temperature of the spinning head was 175 °C and 2700 rpm was used. With these setup parameters, the yield was 73.8 ± 3.9%. The measured drug content of the fibers was 97.8 ± 3.3% of the theoretical value, and the distribution within the fibrous mat was found to be uniform, which is important for later formulations. Furthermore, it was found that the ethanol treatment did not affect the drug content, as the measured values remained practically unchanged (101.6 ± 4.3%).

### 3.2. Microscopic Analysis

The results of fiber diameter analysis of the freshly prepared and the ethanol-treated samples prepared from different types of isomalt are demonstrated in Table 5. The average fiber diameter was 13.44 µm ± 3.61 µm for galenIQ^TM^ 720 (1:1 ratio of GPS:GPM-based fibers), and 15.02 µm ± 6.97 µm for galenIQ^TM^ 721 (3:1 ratio of GPS:GPM).

**Figure 4:**
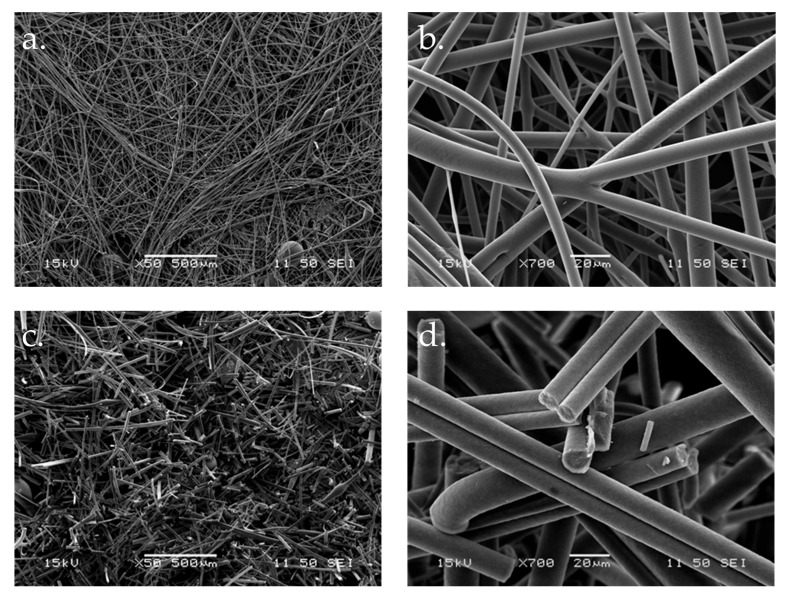
SEM images of solely isomalt (1:1; GPS:GPM; galenIQ^TM^ 720) fibers before (**a**,**b**) and after (**c**,**d**) ethanol treatment (magnification: 50× and 700×).

The fiber diameter of samples stored over ethanol vapor was 12.26 µm ± 5.19 µm in the case of galenIQ^TM^ 720, while the fiber diameter analysis could not be performed for galenIQ^TM^ 721 fibers treated over ethanol vapor owing to their liquefaction upon storage in an alcohol vapor-saturated exsiccator. The change in the fibrous structure of galenIQ^TM^ 720 due to ethanol vapor is shown in the electron microscopic images in Figure 4.

**Figure 5:**
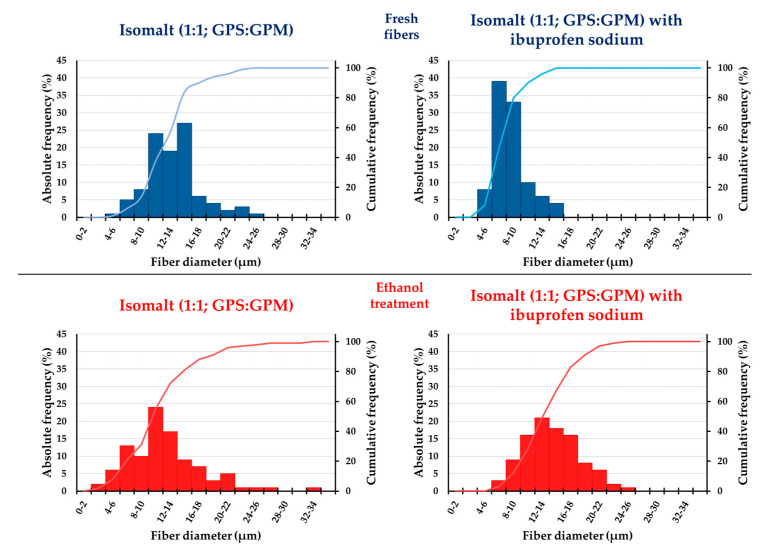
Frequency distribution of fiber diameters for freshly prepared and ethanol-treated fibers composed of isomalt alone, and isomalt combined with ibuprofen sodium; *n* = 100.

Fibers based on galenIQ^TM^ 720 containing the active ingredient behaved similarly to fibers containing only 1:1 GPS:GPM. They retained the fibrous structure, but in a fragmented form. The frequency distribution of fiber diameters was analyzed for freshly prepared and ethanol-treated fibers composed solely of isomalt, as well as for those incorporating both isomalt and ibuprofen sodium (Figure 5).

### 3.3. Macroscopical Morphology Monitoring

**Figure 6:**
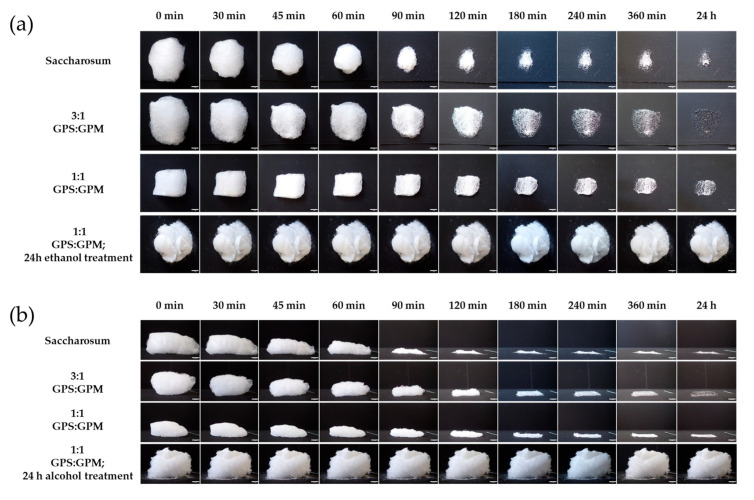
Photographic tracking (scale bar: 1.0 cm) of the collapse behavior of various fiber bundles under ambient conditions (26.5 °C temperature, 44.5% RH): (**a**) top view, (**b**) side view.

The photos (Figure 6) show that the untreated isomalt fibers behave similarly in ambient conditions to cotton candy made from sucrose, and the structure was observed to collapse within the first hour by absorbing the moisture in the air. In contrast, the galenIQ^TM^ 720 isomalt fibers treated with ethanol vapor did not show any visually observable change in the bulk structure even after 24 h, while resisting the damaging effects of environmental moisture on the fibrous structure.

**Figure 7:**
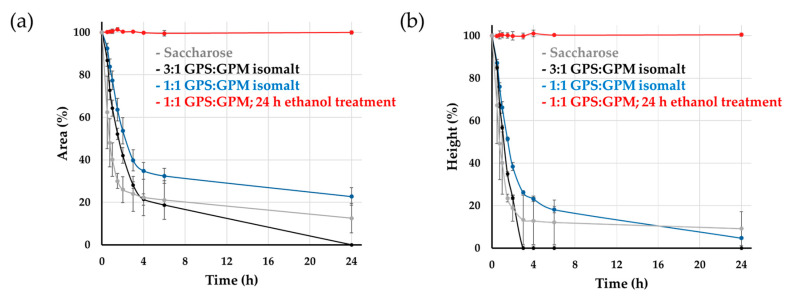
Investigation of fiber bundle behavior under ambient conditions using image analysis: (**a**) surface area; (**b**) height; *n* = 3.

The measurement results obtained from the images are illustrated in Figure 7, which provides numerical evidence of the collapse rate and demonstrates that the treated fibers remained virtually unchanged during the observation period.

### 3.4. Differential Scanning Calorimetry (DSC) Measurements

**Figure 8:**
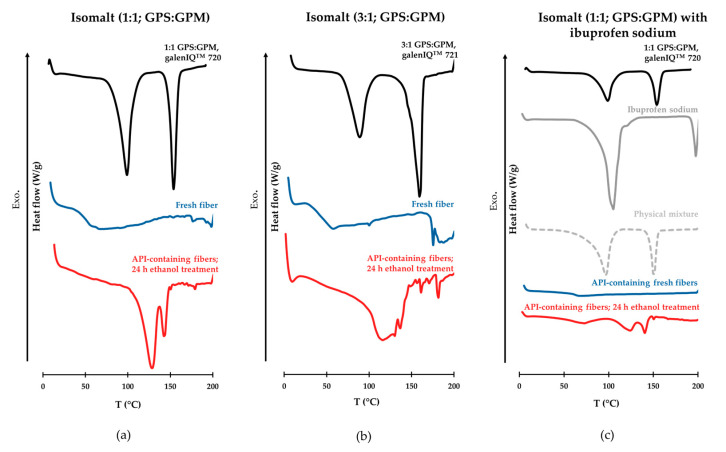
DSC thermograms of raw excipients (black), drug (grey), physical mixture (grey; dotted), and fresh (blue) and ethanol-treated fibers (red); (**a**) 1:1 ratio of GPS:GPM, (**b**) 3:1 ratio of GPS:GPM; (**c**) fibers containing ibuprofen sodium.

Figure 8 presents the DSC thermograms. The thermograms of isomalt raw materials—prepared with varying GPS:GPM ratios—exhibit sharp endothermic peaks, which are indicative of a crystalline structure. Similar distinct peaks are also observed in the thermograms of the active pharmaceutical ingredient (API) and the powder premix containing ibuprofen sodium. In contrast, these sharp crystalline peaks are absent in the thermograms of the freshly prepared fibers made from both types of isomalt and in those containing the API. Instead, a signal corresponding to the glass transition temperature (Tg) of isomalt is observed. However, in the ethanol-treated samples, signs of crystallization appear in the thermograms of all three fiber compositions.

### 3.5. X-Ray Diffraction (XRD) Measurements

**Figure 9:**
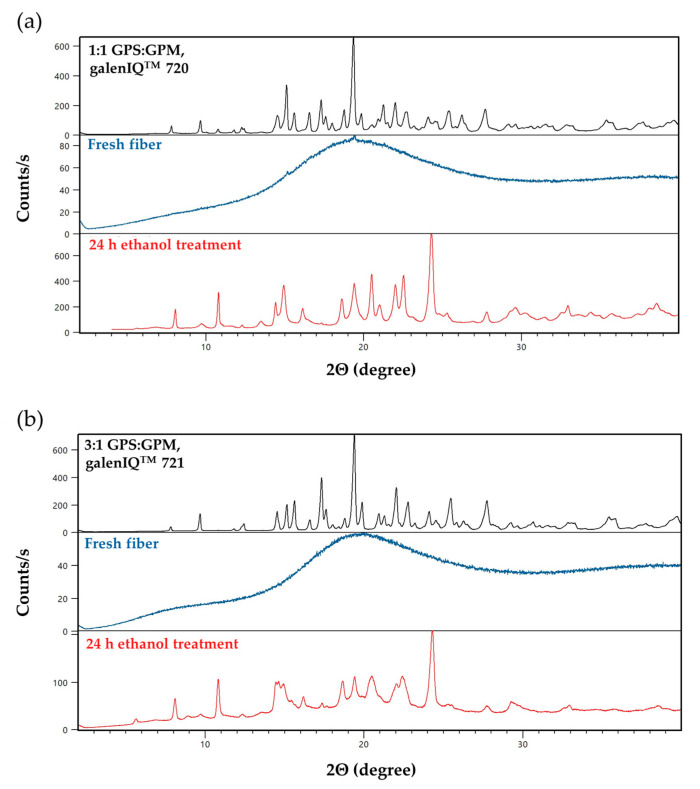
The XRPD patterns of the raw materials (black curves), fresh fibers (blue curves), and ethanol-treated fibers (red curves) prepared from (**a**) 1:1 ratio of GPS:GPM and (**b**) 3:1 GPS:GPM.

**Figure 10:**
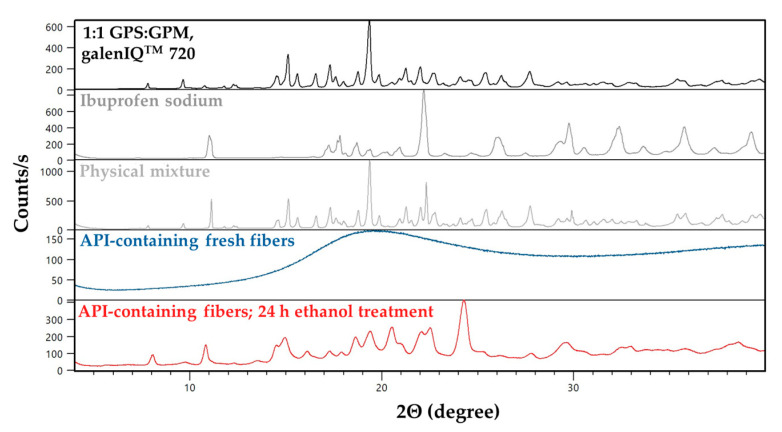
The XRPD patterns of the raw materials (black curve and grey curves), fresh fibers (blue curve), and ethanol-treated fibers (red curve) prepared from 1:1 ratio of GPS:GPM.

The diffractograms obtained from the XRD measurements are shown in Figure 9 and Figure 10.

The results of the DSC measurements were confirmed by the XRPD data for fibers containing the active substance. In the physical powder mixture, distinct reflections of the initial crystalline components are visible. As a result of fiber formation, an amorphous state was formed, as indicated by the absence of sharp peaks. Upon ethanol treatment, recrystallization occurred: peaks characteristic of ibuprofen sodium were observed, along with new peaks differing from those of the initial isomalt.

### 3.6. In Vitro Drug Release Test

**Figure 11:**
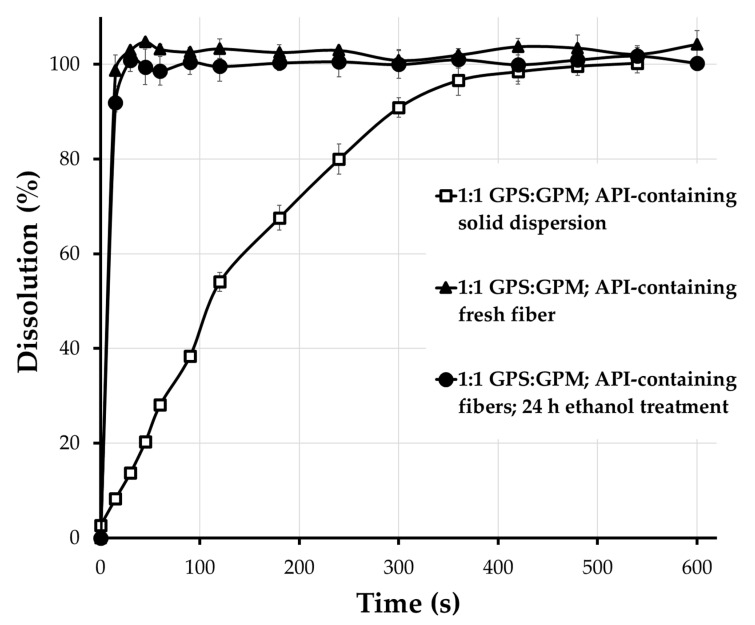
In vitro dissolution test results (mean ± SD; *n* = 3).

Figure 11 presents the drug release profiles of the isomalt-based solid dispersion containing ibuprofen sodium (with a 1:1 GPM:GPS ratio) as well as the freshly prepared and ethanol-treated fibers. It can be clearly observed that nearly the entire amount of the active pharmaceutical ingredient was released from the solid dispersion within 10 min. In the case of drug-loaded fibers, more than 90% of the ibuprofen sodium was released at the first sampling point (15 s) for both the freshly prepared (average 98.69%, SD: 3.30) and the ethanol-treated fibers (average 91.93%, SD: 2.02). As shown in the figure, the standard deviation values remained consistently low throughout the experiment.

## 4. Discussion

Currently, numerous research groups are engaged in the development of fiber formation techniques and the design of oral drug delivery systems utilizing these fibers. Such systems include orodispersible tablets/films, medicated straws filled with drug-loaded fibers, and fiber-filled capsules [40,41,42,43]. An experimental design was developed for the melted fiber formation process, where the effect on yield of two independent variables (temperature and speed of the heatable spinning head) as critical parameters was investigated. Using the examined setting parameters, it was possible to produce a fibrous structure with different weights, similar in appearance to conventional cotton candy. The response equations (Equations (3) and (4)) obtained from the statistical analyses and the surface response curves (Figure 3) clearly demonstrate that in the case of fiber formation from isomalt with different GPS:GPM ratios, the shape of the curves and the order of magnitude of the variables in the equations are similar. The coefficient values of the main effects are statistically significant in both cases (galenIQ™ 720 and 721), indicating that within the investigated experimental range, both the applied setting temperature and the rotational speed have a significant effect on fiber yield. It can be stated that the signs of the coefficients show that, within the tested range, a decrease in temperature and an increase in rotational speed have a favorable effect on the yield. The nonlinear quadratic coefficients (b_11_, b_22_), which represent secondary effects, do not influence the yield under the examined conditions for either material. The combined effect of the two independent variables is not significant, as indicated by the non-significant coefficient values of the interaction parameter (b_12_). Using the results of the statistical analysis, we were able to produce a good yield of isomalt-based (1:1; GPS:GPM) fibers containing the active ingredient. The total amount of the active ingredient was measured in the fibers, which showed a homogeneous distribution.

The diameter of the freshly prepared fibers formed from isomalt alone and the diameter of the fibers containing the active substance were all in the micrometer size range. Alfassam et al. prepared sucrose-based fibers containing miconazole at high temperatures (150–180 °C) by centrifugal spinning. The average diameter of the drug-loaded fibers was found to be 6.6 ± 2.5 μm. In comparison, the average diameter of isomalt-based fibers consisting only of excipients was larger, while the average diameter of isomalt-based fibers containing ibuprofen sodium was found to be in a practically similar range to those measured by Alfassam et al. [44].

In the previously described and patented FlashDose^®^ technology, ethanol was sprayed directly onto melt-formed sucrose or dextrose, lactose, and fructose-based fibers. It is important to note that although ethanol is commonly used in the pharmaceutical industry as a solvent and processing excipient, it may cause problems regarding stability, residual solvents, and equipment compatibility. Its flammability also presents safety risks during manufacturing. Additionally, alcohol-containing formulations may not be suitable for certain patient populations, such as children or individuals with alcohol sensitivity. From a regulatory perspective, ethanol is included in the FDA Inactive Ingredient Database and is approved for use in licensed non-parenteral and parenteral medicinal products in the United Kingdom [45,46].

In the present study, the isomalt-based fibers, due to their large surface area and loose structure, were subjected to ethanol vapor storage. Treatment with ethanol vapor did not cause a considerable change in fiber diameter, where the ratio of GPS to GPM in the isomalt was the same. The galenIQ^TM^ 720 (1:1; GPS:GPM) fibers retained their fibrous structure even after the alcohol treatment was applied. However, the ethanol-treated fibers were fragmented, as was the case with fibers produced with the FlashDose^®^ technology. This suggests that storage in saturated ethanol vapor can induce ethanol atomization on the fiber surface. This fragmentation is clearly visible in the scanning electron microscope images shown in Figure 4. Fragmented fibers are easier to process in pharmaceutical technology. Fiber balls do not have adequate flow properties, so encapsulation or compression is preceded by a grinding process. Numerous publications describe the milling/grinding of fibers prior to tableting [40,47,48]. This step can be avoided or made much simpler by fragmenting the fibers.

Labuza et al. thoroughly investigated the stability of conventional sucrose-based cotton candy at room temperature under various relative humidity conditions. Their results showed that cotton candy stored at room temperature at approximately 0% or 11% RH remained in an amorphous state for at least 2 years. In contrast, when stored at 33% RH, the cotton candy collapsed and crystallized within 3 days. At higher relative humidities, the transformation occurred more rapidly. The samples stored at 45% and 54% RH became rubbery and structurally collapsed in less than an hour, and after 5–6 h, they appeared hard and crystalline [49]. Our observations on sucrose-based cotton candy are consistent with the findings of Labuza. As shown in Figure 6, for the sample stored at 45% RH and 26 °C, structural collapse begins within 1 h, which is clearly visible to the naked eye. After 1 day, the material becomes hard and crystalline. The image analysis results provide quantitative confirmation of the visual observations.

In both cases, the DSC curves of the raw materials reveal the crystalline structure of the isomalt used. The thermograms of various types of isomalt exhibited two endothermic peaks. The first, broader peaks (at about 100 °C) on the thermogram indicate the loss of crystal water, while the second, sharper peaks at approximately 152 °C and 159 °C correspond to the melting points (T_m_) of the samples. The crystal water content of isomalt is linked to the GPM component, as one mole of this component crystallizes with two moles of water, while GPS is present in anhydrous form. This explains why the endothermic peak is larger in the isomalt with a 1:1 GPS:GPM ratio than in galenIQ^TM^ 721, which contains less GPM. The higher melting point is associated with the GPS-richer isomalt type. The measured values are consistent with data reported in the literature [50]. Previous publications have focused mainly on cotton candy made from sucrose. During the fiber formation process, an amorphous state is produced due to rapid cooling and dehydration [51]. This phenomenon was also observed in the case of isomalt, as shown in the thermograms. The sharp peaks characteristic of the crystalline state disappeared, and only the glass transition temperature was observed. For the Tg values characterizing the amorphous state of the freshly prepared isomalt fibers, we observed the trend reported in the literature. Koskinen et al. studied isomalt mixtures with different GPS:GPM ratios (3:1; 1:1) as well as pure GPM in the context of the lyophilization process. Their results showed that the 1:1 GPS:GPM isomalt mixture exhibited the highest physical stability in the amorphous form during freeze-drying. The authors attributed this favorable stability to the stabilization of molecular interactions, which has also been observed in other excipient blends, where the 1:1 ratio was found to be the most stable. This mechanism is presumed to apply to isomalt as well. The 3:1 GPS:GPM isomalt (galenIQ™ 721) exhibits a lower Tg than the 1:1 GPS:GPM isomalt (galenIQ^TM^ 720) [52]. However, new sharp peaks appeared on the DSC curves of the isomalt fibers treated with ethanol vapor, which had undergone the recrystallization process. These peaks are located at positions different from those of the original raw material, and differences can also be observed between the ethanol-treated samples prepared from the two different types of starting isomalt. The same phenomenon can be observed in fibers containing the active substance as in fibers formed from isomalt alone. The thermogram of ibuprofen sodium dihydrate is consistent with that reported in the literature [53].

The results of the DSC measurements were confirmed by PXRD studies. It can be seen that, for the starting raw materials (black curves), several sharp peaks appear in the diffractograms relating to the crystalline form of the isomalts, but, for the freshly prepared fibrous samples (blue curves), the characteristic peaks of the starting material no longer appear. These results indicate that freshly prepared fibers are in amorphous form.

Samples treated with ethanol also show sharp peaks, but their position and intensity differ from the initial isomalts. As with the results of the DSC measurements, it can also be noted that there is a difference between the galenIQ^TM^ 720 and 721 ethanol-treated samples, indicating their different crystal structures. This phenomenon may be due to a change in the crystal structure during ethanol treatment, whereby the amorphous state is not restored to the initial isomalt crystal structure, but a different type of crystal structure is formed.

The PXRD results are in good agreement with the findings of Perkkalainen et al., who successfully prepared GPM crystallized with ethanol, in which the 2:1 ratio of GPM to ethanol was determined by single-crystal X-ray diffraction measurements, and the thermal behavior of the new crystal was investigated by DSC and TG-FTIR (Fourier transform infrared spectroscopy) measurements [54]. Our measured DSC and powder X-ray diffraction results showed that an isomalt was formed with a stable crystal structure and no crystal water. Based on the absence of ethanol signals in the DSC curve, it is most likely that ethanol controlled the crystallization but did not remain in the structure in sufficient amounts to be detected by DSC; however, the new PXRD peaks suggest a new crystal structure formed both for fibers prepared from isomalt alone in the amorphous state with a fibrous structure and for fibers containing the active ingredient.

According to the dissolution results shown in Figure 11, the isomalt base enabled rapid release of the active ingredient even from the solid dispersion. In the case of the fibers, due to their large specific surface area, ibuprofen sodium was released within just a few seconds. Alcohol treatment did not affect the dissolution profile of the drug-loaded fibers.

## 5. Conclusions

Nowadays, research is focused on fiber formation and the development of oral dosage forms utilizing these fibers. Orodispersible systems (such as tablets and strips), medicated straws filled with fibers, and fiber-filled capsules can be produced from these fibers. In this study, isomalt-based fibers with diameters in the micrometer range were successfully produced. A three-level factorial design with two independent variables was employed for the melt-spinning process, revealing a correlation between the critical setting parameters (temperature and speed) and the fiber yield. Due to the resistance of the isomalt fiber structure to environmental factors, recrystallization was carried out. In contrast to FlashDose^®^ technology, however, no alcohol was sprayed onto the fiber surface; instead, ethanol vapor was used during storage. This treatment caused fiber fragmentation, which is advantageous for processability. Fiber processability remains a highly critical issue, as fiber mats often require grinding during tableting or other filling operations, such as capsule or medicated straw production. Fiber fragmentation offers formulation-based benefits by simplifying or potentially eliminating the milling step. The fibrous structure remained stable under environmental stress. Based on the measurements and tests conducted, it can be concluded that this novel isomalt-based fibrous carrier system developed for drug molecules has potential in the formulation of innovative pharmaceutical dosage forms. Due to isomalt’s sweet taste and excellent aqueous solubility, this system is particularly suitable for the development of orodispersible tablets. These attributes not only improve patient compliance—especially among pediatric and geriatric populations—but also support easy administration without the need for water, making the formulation ideal for patients with dysphagia.

## Figures and Tables

**Table 1:** Physicochemical properties of the excipients used in this study based on Ref. [35].

Property		galenIQ^TM^ 720	galenIQ^TM^ 721
Type		agglomerated	agglomerated
GPS:GPM ratio		1:1	3:1
Solubility in water at 20 °C (g/100 g)		25	42
Particle size distribution (µm)	d_10_	95	90
	d_50_	200	180
	d_90_	350	360
SPAN		1.3	1.5
Bulk density (g/dm^3^)		400	400
Tapped density (g/L) *n* = 1250		448	448
Hausner ratio		1.12	1.12
Carr index		10	10
Angle of repose (°)		33	31
Flowability (s/100 g; orifice d = 6.0 mm)		55	57

**Table 2:** The values and codes of the different process parameters.

Coded Value	Actual Value *x*_1_ (Temperature; °C)	Actual Value *x*_2_ (Rotational Speed; rpm)
−1	150	1500
0	175	2100
+1	200	2700

**Table 3:** Mean yields (*n* = 3) and standard deviations of isomalt fibers produced with different GPS:GPM ratios by melt-spinning.

Trial No.	Coded Value of *x*_1_	Coded Value of *x*_2_	Yield (%)	
			1:1; GPS:GPM	3:1; GPS:GPM
1	−1	−1	0.0 ± 0.0	0.0 ± 0.0
2	0	−1	4.8 ± 2.1	4.5 ± 2.2
3	+1	−1	0.0 ± 0.0	0.0 ± 0.0
4	−1	0	51.0 ± 9.4	21.5 ± 9.7
5	0	0	32.9 ± 3.8	21.2 ± 2.0
6	+1	0	6.8± 3.8	0.5 ± 0.4
7	−1	+1	63.2 ± 3.9	59.1 ± 5.2
8	0	+1	64.7 ± 6.0	62.2 ± 5.0
9	+1	+1	13.3 ± 3.0	18.7 ± 3.1

**Table 4:** Polynomial model coefficients and statistical data based on the results of 2^3^ experimental design.

Ratio of GPS:GPM	Model *F*-Value	Parameter	Coefficients					
			*b*_0_	*b*_1_	*b*_2_	*b*_11_	*b*_22_	*b*_12_
1:1	9.864 *p > 0.044*	Value	38.061	−15.692	22.708	−11.772 ^+^	−5.872 ^+^	−12.500 ^+^
		Std. dev.	7.901	4.327	4.327	7.496	7.496	5.300
		*p* > |t|	0.017	0.036	0.013	0.214	0.491	0.100
3:1	24.564 *p > 0.014*	Value	22.858	−10.245	22.562	−12.682 ^+^	9.668 ^+^	−10.103 ^+^
		Std. dev.	4.789	2.623	2.623	4.543	4.543	3.213
		*p* > |t|	0.017	0.030	0.003	0.068	0.123	0.051

^+^: not significant.

**Table 5:** Average diameter of fibers tested (mean ± standard deviation; *n* = 100).

Sample	Storage Condition	Avg. Diameter ± SD (µm)
1:1 GPS:GPM (galenIQ^TM^ 720)	Fresh	13.44 ± 3.61
	24 h ethanol treatment	12.26 ± 5.19
3:1 GPS:GPM (galenIQ^TM^ 721)	Fresh	15.02 ± 6.97
Ibuprofen sodium-loaded fiber	Fresh	8.60 ± 2.32
	24 h ethanol treatment	14.30 ± 3.79

## Data Availability

The data presented in this study are openly available in the article.
